# Early Manifestations of Granulomatosis With Polyangiitis: A Case Report

**DOI:** 10.7759/cureus.116734

**Published:** 2026-09-22

**Authors:** Christian Castano, Rusul Alani, Elizabeth Dockery

**Affiliations:** 1 Family Medicine, Lincoln Memorial University-DeBusk College of Osteopathic Medicine, Knoxville, USA; 2 Family Medicine, Ross University School of Medicine, Bridgetown, BRB; 3 Family Medicine, Covenant Health System, Knoxville, USA

**Keywords:** : acute kidney injury, anemia of chronic disease (acd), granulomatosis with polyangiitis (gpa), migratory arthralgia, pauci-immune glomerulonephritis (gn)

## Abstract

Granulomatosis with polyangiitis (GPA), a rare autoimmune disease formerly known as Wegener’s granulomatosis, is characterized by systemic necrotizing vasculitis that primarily affects small- to medium-sized vessels. Classically, GPA manifests in the respiratory tract and kidneys; however, its early symptoms can be nonspecific, complicating timely diagnosis. This report describes the case of a 43-year-old Caucasian male presenting with progressive polyarthralgia, intermittent fever, weight loss, and other systemic symptoms over three months. Initial clinical findings, including night sweats, fatigue, and a dry cough, were nonspecific and prompted further diagnostic investigations. His condition progressed with worsening anemia, declining kidney function, and elevated inflammatory markers. Diagnostic evaluation, including serologic testing and renal biopsy, ultimately confirmed the diagnosis of GPA with pauci-immune crescentic glomerulonephritis. This report underscores the importance of comprehensive history-taking, thorough physical examination, and a systemic approach to clinical evaluation. It highlights the critical role of primary care physicians in recognizing subtle, systemic symptoms that may point to an underlying autoimmune disease and coordinating early referrals for appropriate diagnostic testing. Timely recognition of GPA can improve outcomes, particularly when appropriate treatment is initiated promptly.

## Introduction

Granulomatosis with polyangiitis (GPA) is a rare systemic necrotizing vasculitis that primarily affects small- to medium-sized vessels. GPA is part of a spectrum of disorders known as antineutrophil cytoplasmic antibody (ANCA)-associated vasculitides, which includes microscopic polyangiitis (MPA) and eosinophilic granulomatosis with polyangiitis (EGPA, formerly known as Churg-Strauss syndrome) [1]. The incidence of GPA is estimated to be 10-20 cases per million people worldwide, and it is more common in Caucasians. The age of onset typically ranges from the fourth to the sixth decades of life, with an approximately 1:1 male-to-female ratio among adults [1].

GPA is commonly associated with positive serologic markers, including cytoplasmic-ANCA (c-ANCA) and PR3 (proteinase 3) antibodies [2,3]. GPA typically involves the upper and lower respiratory tracts and kidneys, leading to manifestations such as sinusitis, pulmonary hemorrhage, and rapidly progressive glomerulonephritis (RPGN) [2,4]. However, its early symptoms can be nonspecific and may include polyarthralgia, malaise, epistaxis, and unexplained anemia, potentially resulting in significant delays in diagnosis [5,6].

This case report emphasizes the critical role of primary care physicians in recognizing subtle systemic symptoms and coordinating timely evaluation and referral for suspected vasculitis. The patient initially presented with nonspecific complaints, including night sweats, fatigue, weight loss, intermittent fevers, and arthralgia [7,8]. The challenges associated with diagnosing an uncommon disease such as GPA are highlighted through the extensive diagnostic workup, including laboratory analyses, imaging, and histopathologic evaluation. Serology demonstrated positive c-ANCA and PR3 antibodies, and a renal biopsy confirmed the diagnosis of pauci-immune crescentic glomerulonephritis.

## Case presentation

A 43-year-old Caucasian male with a medical history notable for iron deficiency anemia (IDA), hyperlipidemia, and allergic rhinitis, with recent corrective surgery for a deviated septum, presented to the family medicine residency clinic with a three-month history of diffuse joint pain. His symptoms included bilateral ankle, wrist, and small joint pain, accompanied by intermittent low-grade fevers, unintentional weight loss, and generalized fatigue. A review of systems (ROS) revealed intermittent fevers, chills, night sweats, nocturia, and a dry, intermittent cough.

The patient denied recent travel, nausea, vomiting, abdominal pain, changes in bowel habits, dysuria, melena, rashes, mood changes, vision disturbances, or generalized pruritus. Review of prior medical records revealed IDA on peripheral blood smear, an unremarkable esophagogastroduodenoscopy (EGD) and colonoscopy, and an unintentional 20-pound weight loss over the preceding three months. His family history was significant for thyroid disease in his mother and cardiovascular disease and hyperlipidemia in his father. His paternal grandfather had a history of esophageal cancer.

On physical examination, his vital signs were as follows: temperature of 36.6°C, heart rate of 75 beats per minute, blood pressure of 147/84 mmHg, oxygen saturation of 97% on room air, weight of 84.8 kg, and BMI of 30 kg/m². The cardiopulmonary examination was unremarkable, and the abdominal examination revealed no abnormalities. Musculoskeletal examination showed tenderness over the bilateral forearms and the left side of the neck below the angle of the mandible, as well as moderate tenderness and mild edema in the bilateral hands and feet. No bony abnormalities or midline spinal tenderness were noted, and no rashes were observed.

Laboratory findings were significant for elevated inflammatory markers, with an erythrocyte sedimentation rate (ESR) of 85 mm/hr and C-reactive protein (CRP) of 150 mg/L. A comprehensive metabolic panel (CMP) showed elevated creatinine of 3.31 mg/dL and a decreased glomerular filtration rate (GFR) of 23 mL/min. Given these concerning findings, including worsening kidney function, the patient was referred to the emergency department (ED) for further evaluation.

ED admission laboratory studies

In the ED, initial laboratory studies revealed a hemoglobin of 8.3 g/dL, hematocrit of 24.7%, mean corpuscular volume (MCV) of 77 fL, and an elevated white blood cell (WBC) count of 11.3 × 10³/μL. Additional studies demonstrated an iron level of 18.7 mcg/mL and a ferritin level of 988 ng/mL, suggestive of combined IDA and anemia of chronic disease. A peripheral blood smear confirmed microcytic anemia. Urinalysis (UA) showed 3+ blood, 2+ protein, and grossly cloudy urine, with microscopic examination revealing over 50 red blood cells (RBCs), granular casts, and 6-10 WBCs. Laboratory results are detailed in Table 1 and Table 2.

**Table 1:** CBC with differential and UA CBC: complete blood count; UA: urinalysis; WBC: white blood cell; RBC: red blood cell; Hgb: hemoglobin; Hct: hematocrit; MCV: mean corpuscular volume; RDW: red cell distribution width; PLT: platelets

	Normal range	Patient value
Total WBC count, × 10³/µL	4-11	11.3
RBC count, × 10⁶/µL	4.7-6.1	3.10
Hgb, g/uL	13.8-17.2	8.3
Hct, %	40-52	24.70
MCV, fL	80-100	77
RDW, %	11.5-14.5	14.50
PLTs, × 10³/μL	150-450	572
Absolute neutrophil, × 10³/μL	1.5-8.0	8.57
Iron level, mcg/dL	65-175	18.7
Ferritin level, ng/mL	30-566	988.0
	Normal values	Patient values
UA blood	None	3+
UA protein	None	2+

**Table 2:** Routine chemistry BUN: blood urea nitrogen; eGFR: estimated glomerular filtration rate

	Normal range	Patient value
Sodium level, mEq/L	135-145	135
Potassium level, mEq/L	3.5-5.0	4.8
Chloride level, mEq/L	96-106	102
CO_2, _mEq/L	22-29	18
Glucose level, mg/dL	70-120	90
BUN, mg/dL	7-20	43
Serum creatinine, mg/dL	0.74-1.35	3.25
Calcium level, mg/dL	8.5-10.5	9.2
Anion gap, mEq/L	8-12	13
eGFR, mL/min/1.73 m²	≥ 90	23
Lactic acid, mmol/L	0.5-1.0	1.0

Given the patient's worsening renal function, fatigue, polyarthralgia, and systemic inflammatory signs, he was admitted to the hospital for further workup and management of acute kidney injury (AKI). Nephrology was consulted, and additional investigations were initiated. Further laboratory studies revealed an elevated rheumatoid factor of 192 IU/mL and positive antinuclear antibodies (ANA) at a titer of 1:80. Cyclic citrullinated peptide (CCP) antibodies were within the normal range. The results of the serologic, immunologic, and autoimmune assays are detailed in Table 3.

**Table 3:** Immunology/serology/autoimmune assay ANA: antinuclear antibody; CCP ab: anti-cyclic citrullinated peptide antibodies; dsDNA: double-stranded DNA; c-ANCA: cytoplasmic anti-neutrophil cytoplasmic antibody; p-ANCA: perinuclear anti-neutrophil cytoplasmic antibody; Hep A/B/C: hepatitis A/B/C

	Patient value
ANA screen	Positive
ANA titer	1:80
C3/C4 complement	140/30 mg/dL
CCP Ab	< 15.6 units (normal: < 20)
dsDNA IgG	< 10 IU/mL (normal: < 30)
c-ANCA	Positive (1:512)
p-ANCA	Negative
RF quant	192 IU/mL (normal: < 14)
Hep B surface Ag	Nonreactive
Hep C antibody	Nonreactive
PR3 antibody	> 26 AU/mL (normal: < 19-20 AU/mL)
Cryoglobulin, S	Negative

Renal imaging and diagnosis

Renal ultrasound showed increased renal parenchymal echogenicity, compatible with nonspecific renal parenchymal disease. These findings were considered potentially consistent with the clinical suspicion of renal involvement in a systemic process, such as vasculitis (Figures 1-3).

**Figure 1:**
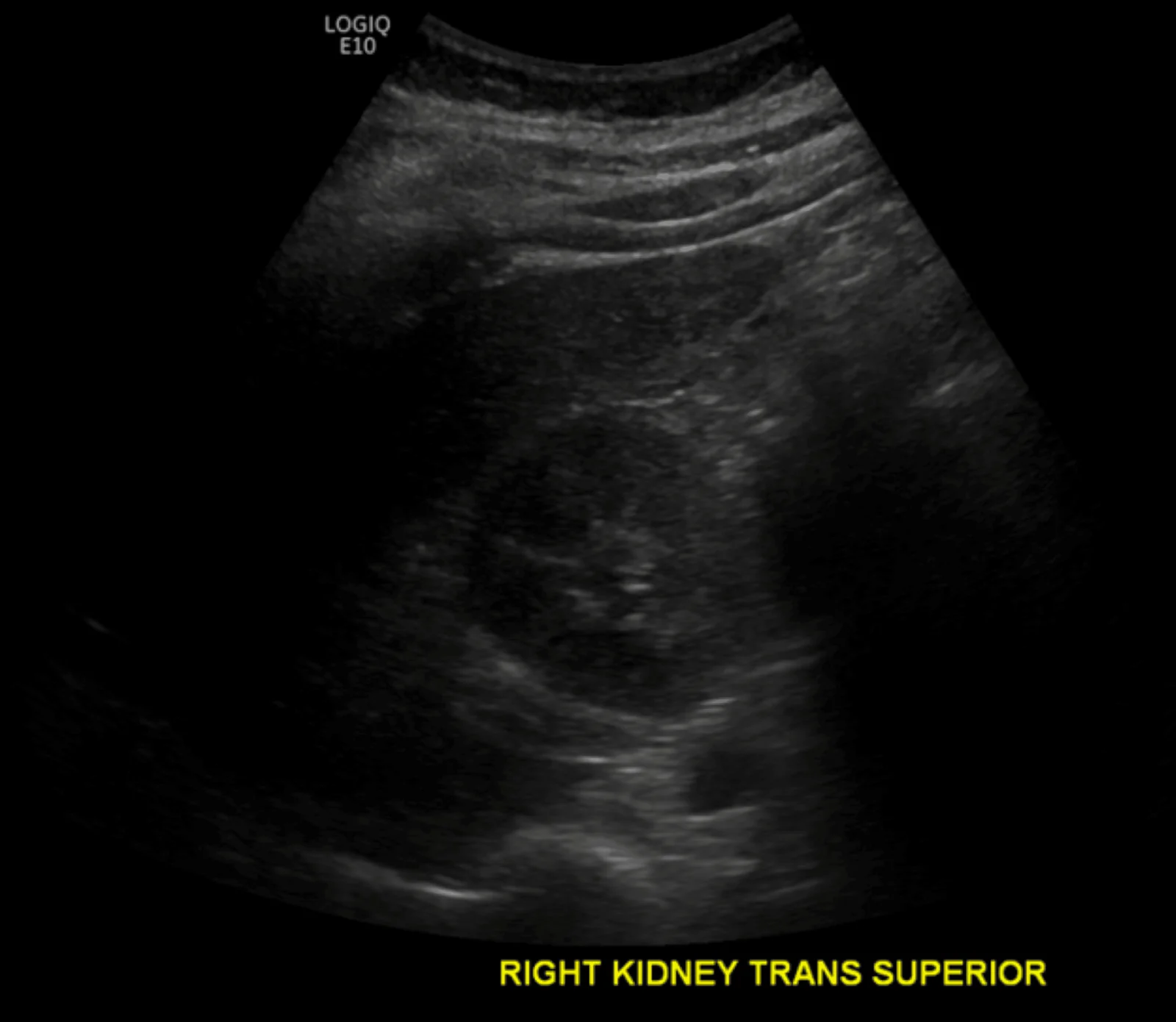
Transverse superior view of the right kidney

**Figure 2:**
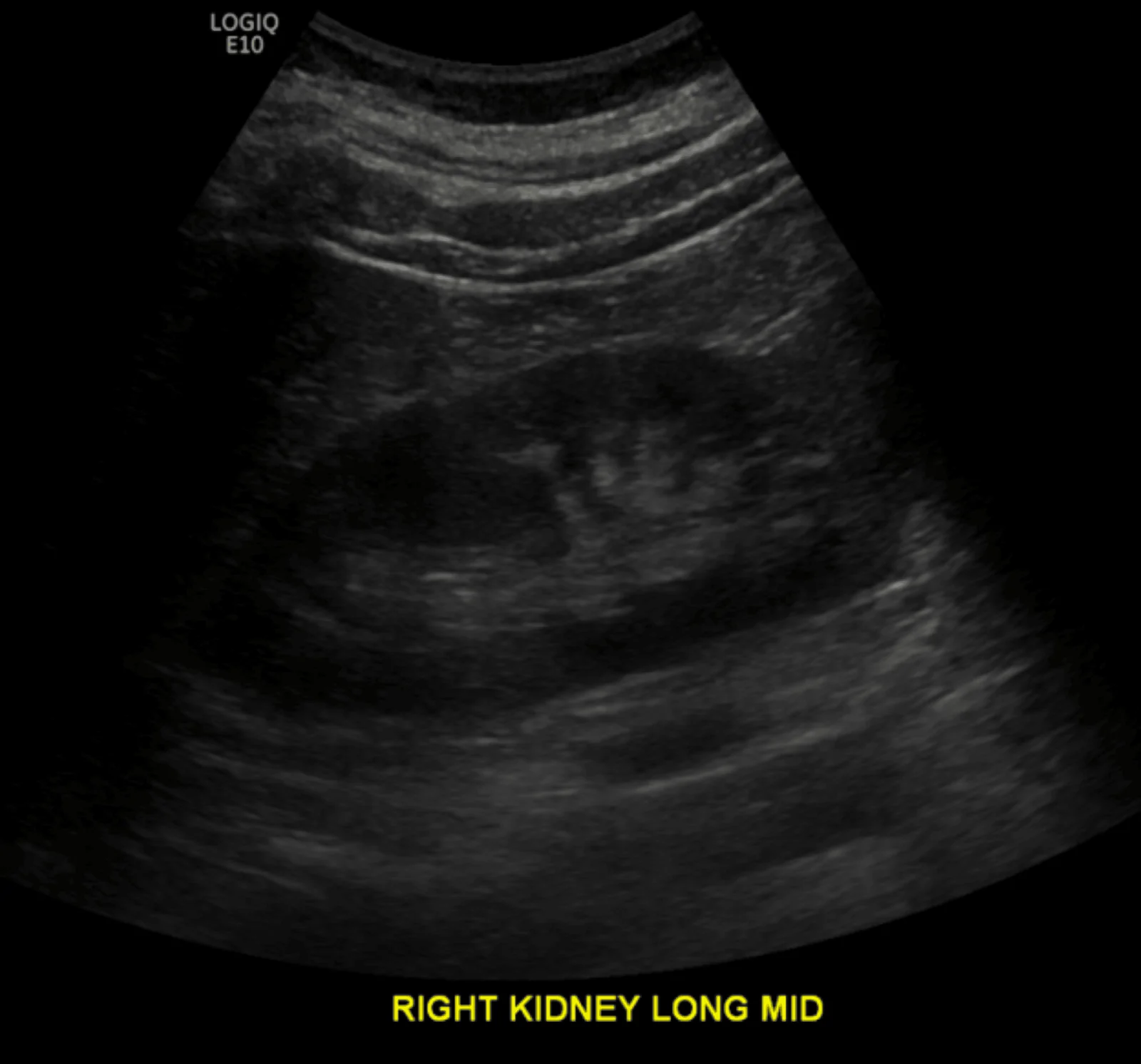
Longitudinal mid view of the right kidney

**Figure 3:**
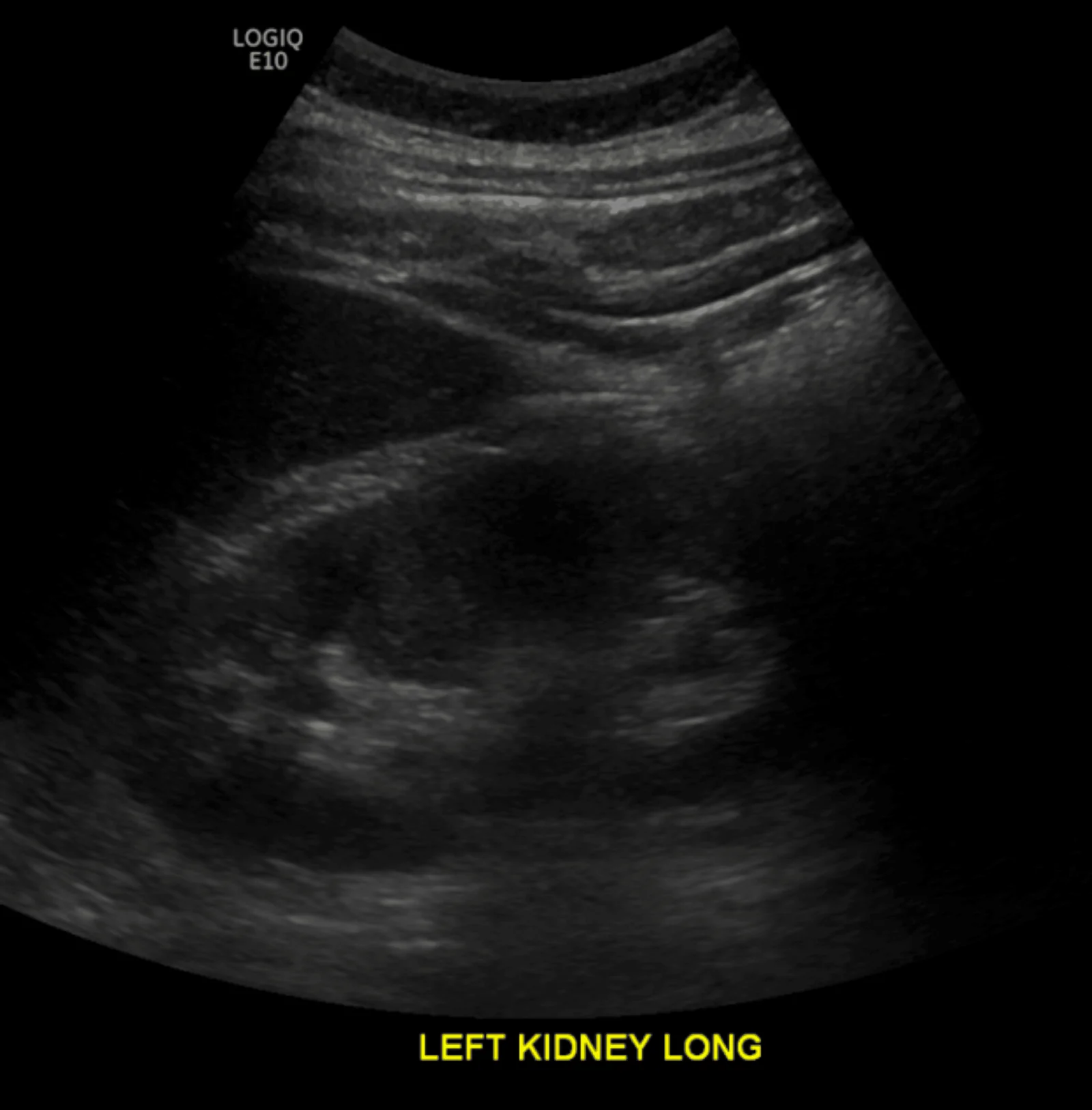
Longitudinal view of the left kidney

Serologic and biopsy findings

Serologic testing was positive for c-ANCA, with a PR3-positive result, supporting a diagnosis of GPA. A renal biopsy was performed and revealed necrotizing crescentic glomerulonephritis, consistent with pauci-immune vasculitis, providing evidence of GPA with significant renal involvement. The pathology report from the kidney core needle biopsy detailed the following findings: pauci-immune necrotizing and crescentic (~70%) glomerulonephritis; acute tubular injury with associated tubulointerstitial nephritis; mild to moderate interstitial fibrosis and tubular atrophy; mild arteriosclerosis (Figures 4-6).

**Figure 4:**
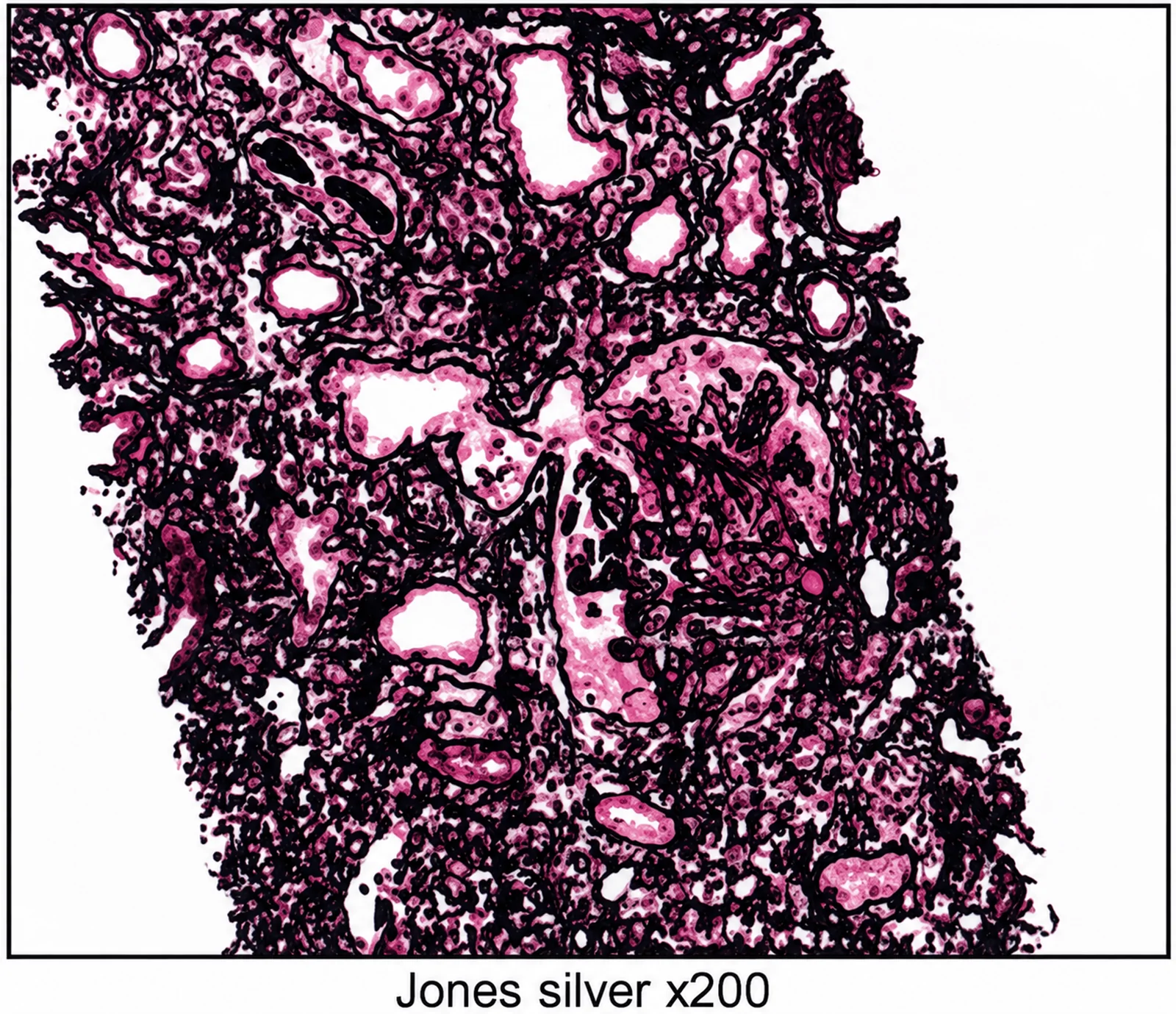
Glomerular light microscopy with Jones Silver stain Sections (H&E x3; PAS x2; trichrome x2; Jones methenamine silver x1; all with appropriate controls) show one core of renal cortex with focal corticomedullary junction and superficial medulla containing up to 15 glomeruli, one of which is globally sclerosed. There is no increase in mesangial matrix or cellularity, and basement membranes display focal segmental ischemic corrugation and contour duplication without the appearance of spikes or holes on silver stains. Eleven glomeruli display segmental fibrinoid necrosis with basement membrane breaks, four of which have associated cellular crescents, and five have fibrocellular crescents. Additionally, one glomerulus displays a fibrocellular crescent without diagnostic necrosis, but there is no endocapillary proliferation. The globally sclerotic glomerulus and one glomerulus with a fibrocellular crescent show rupture of Bowman’s capsule with surrounding interstitial inflammation. There are diffuse dense lymphoplasmacytic interstitial inflammatory infiltrates with frequent eosinophils and scattered neutrophils, and there is mild tubulitis of injured and early atrophic tubules. Interstitial fibrosis and tubular atrophy are estimated to be ~20-30%. Evaluable nonatrophic tubules are diffusely dilated with epithelial flattening, apical blebbing, loss of brush borders, and sloughing. There are occasional proteinaceous, granular, and focal red blood cell casts, but there are no crystals or polarizable material. Arterioles show mild to moderate medial thickening, and there is mild intimal fibrosis and medial thickening of small arteries without evidence of vasculitis or thrombotic microangiopathy. Larger arteries are not sampled H&E: hematoxylin and eosin; PAS: periodic acid–Schiff

**Figure 5:**
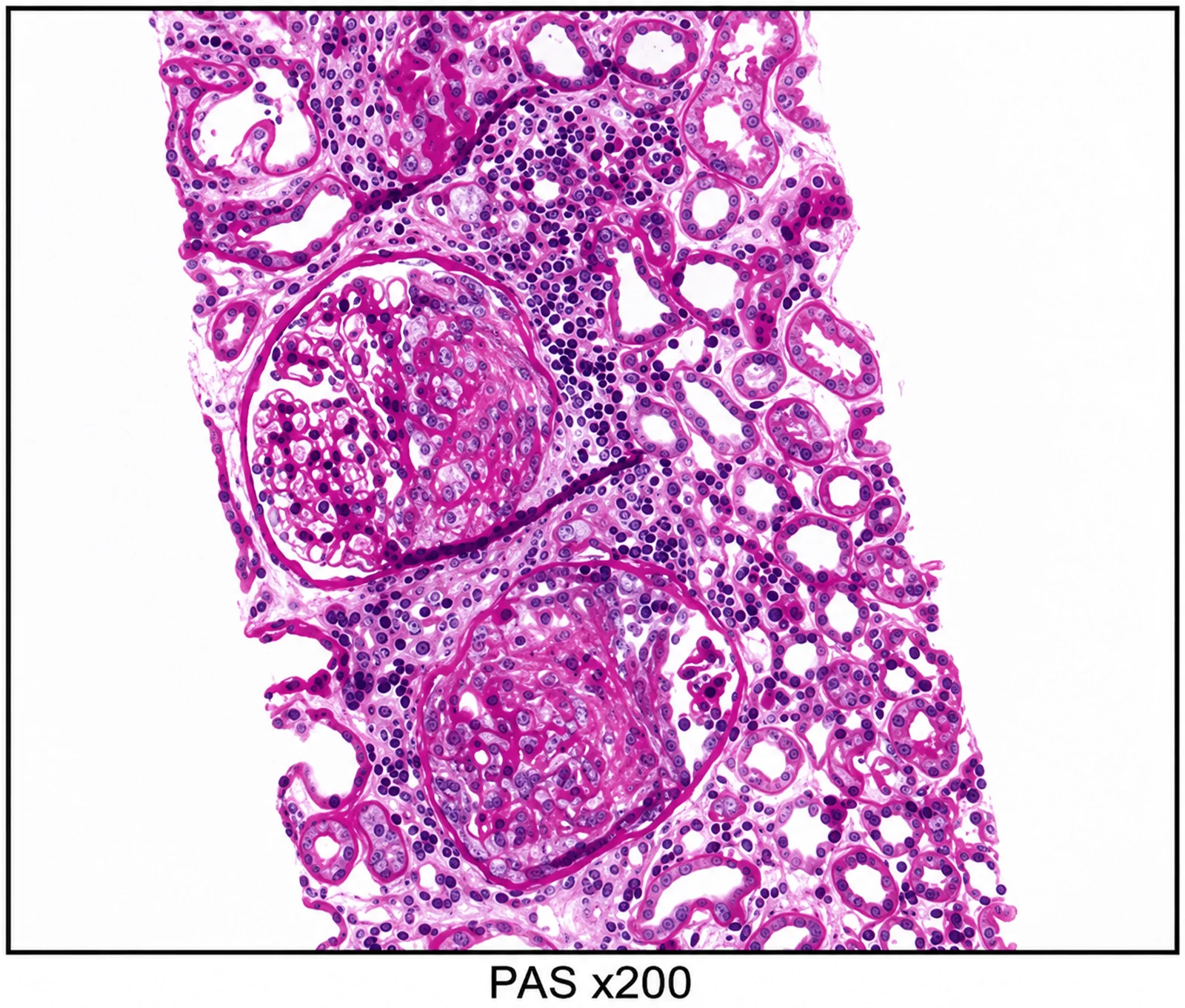
Glomerular light microscopy with PAS stain Frozen sections containing 13 glomeruli, one of which is globally sclerosed and at least five of which show associated necrosis and basement membrane breaks, are stained with fluorescein antisera to IgG, IgA, IgM, C3, C1q, kappa, lambda, albumin, and fibrinogen. There is focal nonspecific segmental glomerular staining in areas of injury for IgG, C3, kappa, and lambda, but there is no diagnostic immune complex-type staining. There is focal 3+ staining for fibrinogen associated with areas of apparent necrosis. Casts stain for IgG (rare 2+), IgA (3+), C3 (rare 3+), kappa (3+), lambda (2+), and albumin (rare 3+), and there is 2-3+ arteriolar staining for C3. There is no diagnostic staining for IgM or C1q

**Figure 6:**
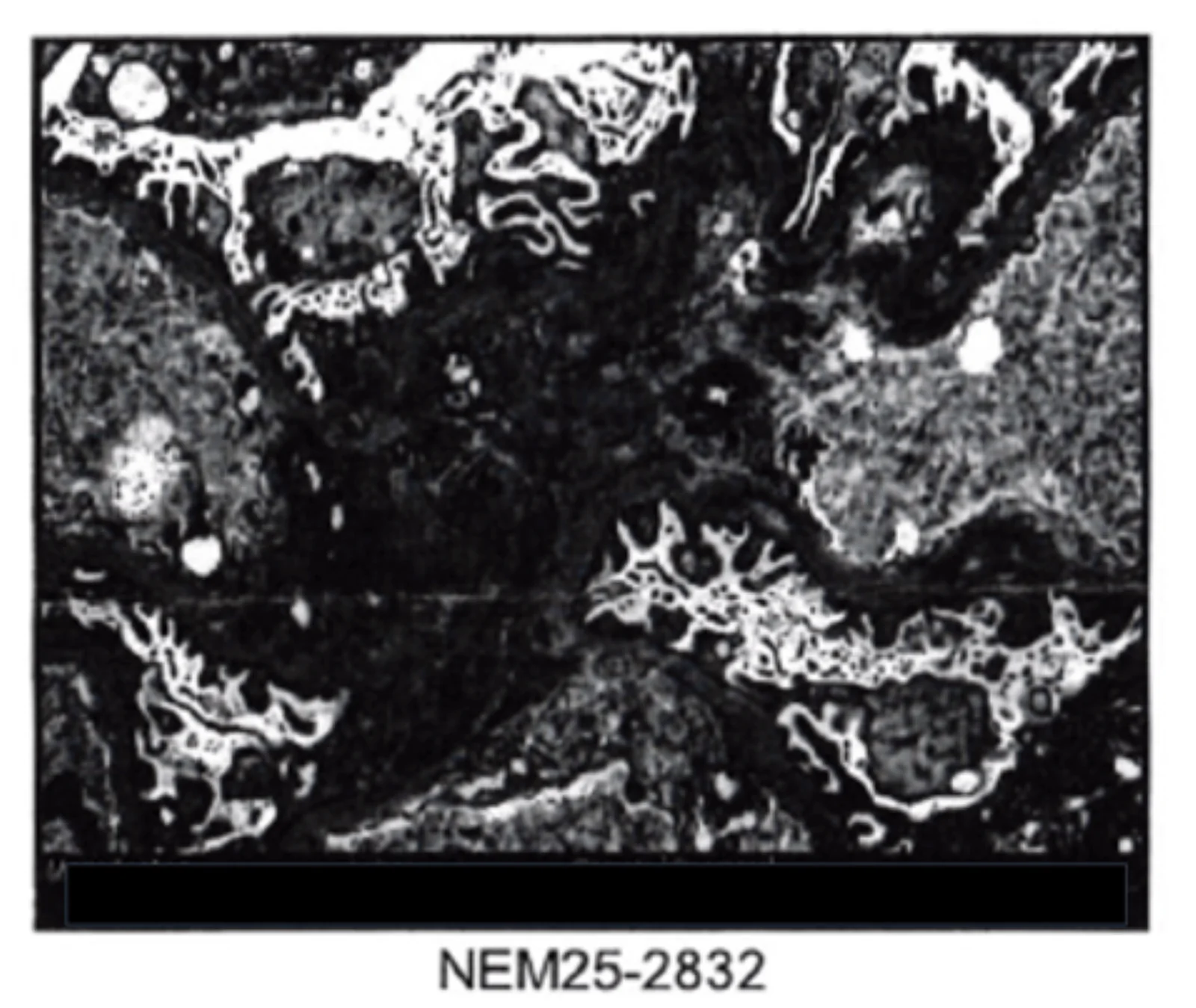
Glomerular electron microscopy Specimen submitted for electron microscopy, studied first by light microscopic examination of toluidine blue-stained one-micron-thick sections, shows fragments of renal cortex and medulla containing three glomeruli, one of which shows segmental fibrinoid necrosis. Morphologic features are similar to those described in light microscopy. Ultrastructural examination reveals portions of capillary loops with patent lumina. Glomerular basement membranes are not thickened, averaging 349 nm, with segmental corrugation and rare cellular interposition. There are no diagnostic immune complex deposits. There is mild (~20%) foot process effacement. Endothelial cells show segmental mild swelling. There are segmental glomerular basement membrane breaks with associated fibrin tactoids. Mesangial matrix is segmentally increased without hypercellularity. There are no tubular basement membrane deposits

Treatment and clinical course [5]

The patient was started on aggressive treatment with the following regimen: intravenous Solu-Medrol (three doses), followed by an oral prednisone taper, and rituximab induction therapy, with the first dose of 1000 mg followed by subsequent doses at two weeks and six months.

The patient's renal function gradually improved during his hospitalization, as reflected in repeat laboratory tests demonstrating a reduction in serum creatinine to 2.48 mg/dL and an improvement in eGFR to 32 mL/min. Hemoglobin and hematocrit levels also improved to 9.5 g/dL and 28.9%, respectively. He was discharged with plans for outpatient follow-up with nephrology, rheumatology, and primary care. At the time of discharge, the patient’s arthralgias and constitutional symptoms had significantly improved. Follow-up at the Family Medicine residency clinic 10 days after discharge showed continued resolution of symptoms, and the patient was tolerating the treatment regimen well.

## Discussion

GPA remains a diagnostic challenge in primary care due to its often nonspecific and variable early manifestations. Patients typically present with constitutional symptoms such as fatigue, weight loss, and fever, alongside arthralgias, which may precede more severe organ involvement by weeks to months. In its early stages, laboratory findings such as anemia and elevated inflammatory markers (e.g., ESR and CRP) are common, but they are nonspecific and may be the only clues before more distinctive symptoms of renal or pulmonary involvement emerge. One common characteristic manifestation of GPA is sinonasal disease, which may be misdiagnosed as other nasal conditions. Septal perforation and saddle-nose deformity resulting from cartilage and bone destruction are hallmark features of GPA's extravascular manifestations. A key distinction from a simple deviated septum is that GPA causes destructive and inflammatory mucosal disease, whereas a deviated septum is a static structural/anatomic variant without inflammation or tissue loss [6].

This case highlights an atypical outpatient presentation of GPA, initially manifesting as nonspecific constitutional symptoms with delayed progression to rapidly progressive glomerulonephritis. The diagnosis was established based on compatible serologic findings and confirmed by renal biopsy demonstrating pauci-immune necrotizing crescentic glomerulonephritis. The etiology of GPA consists of several complex interactions resulting in defective immunoregulatory responses. This consequently leads to excessive production of cytokines, causing inflammatory granulomatous vascular lesions [1]. The two primary types of ANCAs are anti-PR3 and anti-myeloperoxidase (MPO). PR3 is positive in approximately 75% of GPA cases, while MPO is positive in about 10% of cases, and fewer than 10% are ANCA-negative [3]. The binding of ANCA to PR3 activates neutrophils, increasing their adherence to the endothelium and triggering degranulation, resulting in damage to endothelial cells [3].

There are genetic predispositions to developing GPA, including defective alleles involving alpha-1 antitrypsin, cytotoxic T-lymphocyte-associated protein 4 (CTLA-4), the PR3 gene, major histocompatibility complex, class II, DP alpha-1 (HLA-DP) gene, and specific Fc gamma receptor IIIb found on neutrophils, macrophages, or monocytes [7]. Bacterial infections, specifically Staphylococcus aureus, as well as viral infections including hepatitis C, CMV, EBV, and parvovirus, have been reported to modulate the manifestation of GPA [7]. Certain medications can also trigger GPA, including immunosuppressive agents such as sulfasalazine, antithyroid medications such as PTU and methimazole, as well as antibiotics such as rifampicin and minocycline [4].

In this case, the patient's initial presentation, characterized by arthralgia and anemia, progressed to kidney involvement, ultimately linked to an underlying autoimmune vasculitis. This highlights the progressive nature of GPA and the importance of recognizing early renal involvement, as GPA-associated glomerulonephritis can rapidly progress to end-stage renal disease (ESRD) if left untreated. The patient's initial bloodwork demonstrated anemia of chronic disease, suggested by an elevated serum ferritin level of 988 ng/mL, as well as chronic IDA with an iron level of 18.7 mcg/mL at the time of admission. Anemia of chronic disease is a hallmark finding in chronic vasculitides, such as GPA.

Primary care physicians play a critical role in detecting systemic inflammatory patterns and initiating timely diagnostic workup, including serologic testing for ANCA (anti-neutrophil cytoplasmic antibodies), which is highly specific (~90%) for GPA, particularly when associated with positive c-ANCA and PR3 specificity [1]. Early identification allows for prompt intervention, which is essential for preventing irreversible organ damage.

The treatment of GPA typically involves aggressive immunosuppression, including high-dose corticosteroids for induction therapy, followed by maintenance therapy with rituximab or azathioprine for 12 to 24 months to prevent relapse and reduce long-term complications. Early recognition of subtle systemic features of GPA is critical, as prompt immunosuppressive therapy may prevent irreversible end-organ damage.

## Conclusions

This case report underscores that GPA can initially present with nonspecific constitutional symptoms or even mimic a benign illness or malignancy. Early recognition and referral by primary care clinicians are essential for improving outcomes, as delayed diagnosis can lead to significant, irreversible organ damage. A systematic and thorough approach to unexplained inflammatory symptoms, anemia, and constitutional complaints is crucial for identifying systemic vasculitis early in its course. In this case, the diagnosis was confirmed through a combination of serological studies, renal biopsy, and clinical findings, including pauci-immune crescentic glomerulonephritis on renal biopsy and positive c-ANCA/PR3 serology, supporting the diagnosis of ANCA-associated vasculitis. The workup, including comprehensive laboratory studies, renal imaging, and multidisciplinary collaboration, emphasized the importance of maintaining an open differential diagnosis and a broad investigative approach. This strategy ultimately led to an accurate diagnosis, highlighting the importance of early, multidisciplinary intervention to provide optimal care for patients.
